# Supplementary material for: The unequal burden of human-wildlife conflict
Source: Commun Biol. 2023 Feb 23;6:182. doi: 10.1038/s42003-023-04493-y (PMC9950466; doi:10.1038/s42003-023-04493-y)
Supplement: Supplementary file 2 — Supplementary Information [file 42003_2023_4493_MOESM2_ESM.pdf]

Supplementary Information

The unequal burden of human-wildlife conflict (13 February 2022)

Supplementary Box 1: Economic definitions underpinning our global assessment of HWC cost disparities resulting from carnivore depredation of cattle (i.e., potential economic burden).

Definitions

Household income - a measure of the combined incomes of all people living in a single household. This includes every form of income including salaries and wages, retirement benefits, near cash government transfers like food stamps, and investment gains. In our analysis we use regional per capita GDP from Lessmann and Seidel (2017) as a proxy for household income. We do this not only due to the lack of detailed information on this variable at a global scale, but also due to the high correlation between per capita GDP and household income. In the absence of significant taxes and transfers, and international capital flows – which is a valid assumption for rural areas in developing countries -- GDP and household income are almost equivalent.

Potential burden - defined in our analysis as direct economic impact on household income. Calculated by dividing the live cattle weight price (per kg, FAO 2021) by the regional per capita income at a given locality. In our analysis this is measured both at the regional (ie. administrative level) and country level.

Area of Habitat (AOH) - represent areas of fine scale (300m) habitat suitability for 18 large carnivore species within their IUCN geographic distributions during the 2009 year (Rondinini et al. 2011).

Caloric cost (direct and lost opportunity) - estimated direct calories lost to a household from the depredation of a single cow or bull. Data are taken from FAO meat yield per animal per country. These calories are divided by the average daily caloric intake of young children aged 2-3, adolescents aged 12-13, and 30-60-year-old adults (UN 2004). Lost opportunity costs are calculated by estimating the potential calories lost from milk and meat production from one lost cattle calf (Otte & Chilonda 2002).

Supplementary Table 1: Species, range extents, threat status and justification for inclusion of 18 large carnivores known to predate on cattle globally.

Species name	Scientific name	IUCN category	Range size (km^2)	Justification for inclusion
African wild dog	Lycaon pictus	EN	979806150	1
American black bear	Ursus americanus	LC	8207057520	2
Asian black bear	Ursus thibetanus	VU	1793466450	3
Brown bear	Ursus arctos	LC	20307693870	4
Brown hyena	Parahyaena brunnea	NT	1986171030	5
Cheetah	Acinonyx jubatus	VU	2426897970	6

Dhole	Cuon alpinus	EN	5219226720	7
Dingo	Canis lupus dingo	LC	6615007920	8
Gray wolf	Canis lupus	LC	27113006880	9
Jaguar	Panthera onca	NT	7006778370	10
Leopard	Panthera pardus	VU	15957731970	11
Lion	Panthera leo	VU	3407351490	12
Puma	Puma concolor	LC	16759354770	13
Snow leopard	Panthera uncia	VU	2022763140	14
Spotted hyena	Crocuta crocuta	LC	8436514590	15
Striped hyena	Hyaena hyaena	NT	3400944300	16
Tiger	Panthera tigris	EN	1469565000	17

Species omitted: Clouded leopard *Neofelis nebulosa*, Eurasian lynx *Lynx lynx*, Maned wolf *Chrysocyon brachyurus*, and Ethiopian wolf *Canis simensis* due to no evidence being found in the available peer-reviewed literature of these species consuming cattle in their diet (18,19).

Supplementary Table 2: Percentage of per capita annual income lost in a single calf depredation event across the range of 18 large carnivores globally. All country-level economy classifications are taken from Guterres (2020), DEV=Developing, TRA=Transition, and DPD=Developed.

**Country**	**Economic classification**	**Lowest % of per capita annual income lost**	**Highest % of per capita annual income lost**	**Mean % of per capita annual income lost**	**Standard Deviation**
Mozambique	DEV	4.18	210.08	201.39	14.08
Cambodia	DEV	7.51	152.73	149.94	7.51
Guinea-Bissau	DEV	62.95	125.16	124.88	1.94
Laos	DEV	7.69	152.73	121.81	9
Malawi	DEV	3.42	210.08	119.48	16.29
Tanzania	DEV	3.42	140.63	116.57	8.85
Uganda	DEV	13.8	118.5	113.96	7.81
Burundi	DEV	44.46	123.84	106.02	8.17
Gambia	DEV	41.6	92.01	88.71	4.52
Angola	DEV	3.84	78.9	77.28	4.22
Lesotho	DEV	66.52	86.47	74.51	7.31
Ethiopia	DEV	0	70.69	69.5	2.09
DRC	DEV	3.84	115.72	66.59	4.82
Rwanda	DEV	44.46	115.48	58.23	7.73
Eritrea	DEV	22.25	70.35	56.67	3.38
Niger	DEV	24.89	54.62	53.74	1.73
Albania	TRA	5.24	59.99	51.5	5.67
Burkina Faso	DEV	28.98	53.78	49.63	2.77
Mali	DEV	19.56	53.78	44.53	1.25
China	DEV	0.44	125.24	42.77	8.3
Myanmar	DEV	3.01	125.01	42.24	4.11
Yemen	DEV	4.88	54.75	40.95	7.08
Guyana	DEV	9.69	42.48	40.8	3.62
Chad	DEV	13.87	54.29	39.63	0.67
Iraq	DEV	0	47.74	39.61	4
Mauritania	DEV	20.24	45.1	37.11	0.24
Armenia	TRA	15.51	43	35.23	4.86
Tajikistan	TRA	24.08	44.49	34.56	7.53
Nigeria	DEV	13.04	54.29	34.12	3.6
Bosnia and Herzegovina	TRA	5.04	34.13	33.52	2.44
Afghanistan	DEV	1.93	44.49	33.32	3.22
Kyrgyzstan	TRA	6.56	44.49	32.23	4.02
Algeria	DEV	0	35.7	29.91	6.5
Vietnam	DEV	17.6	152.7	26.58	5.88
Djibouti	DEV	15.71	70.69	26.09	3.16
Congo	DEV	18.11	47.68	25.89	2.29
Honduras	DEV	0	32.61	25.78	4.8
Turkey	DEV	0	42.93	25.42	1.98

Benin	DEV	15.47	50.75	25.2	1.46
Guinea	DEV	13.35	25.77	25.14	0.89
Mongolia	DEV	11.48	44.49	25.08	1.25
Zimbabwe	DEV	13.21	27.15	25.06	1.45
Morocco	DEV	1.43	29.2	24.5	8.12
Sudan	DEV	14.28	116.37	24.41	2.7
Equatorial Guinea	DEV	23.98	24.01	23.99	0.01
Ivory Coast (Coted'Ivoire)	DEV	15.85	27.16	23.46	2.78
Botswana	DEV	4.48	26.35	23.35	2.11
Togo	DEV	15.47	22.95	21.75	1.31
Indonesia	DEV	16.68	26.75	21.46	2.56
Sierra Leone	DEV	0	21.4	21.11	1.07
Egypt	DEV	12.02	24.18	20.89	1.78
Bhutan	DEV	2.37	56.35	20.87	2.99
Uzbekistan	TRA	1.96	31.91	20.58	2.39
Palestine	DEV	6.27	20.92	20.35	2.82
Senegal	DEV	9.41	125.16	19.95	1.44
Georgia	TRA	8.58	22.85	18.15	1.93
Republic of Moldova	TRA	7.54	19.5	18.09	3.86
Suriname	DEV	8.16	41.1	18.04	1.97
Azerbaijan	TRA	9.48	43	17.56	3.42
Kenya	DEV	0	116.37	17.44	3.07
Bangladesh	DEV	2.37	47.52	17.21	1.05
Iran	DEV	1.97	44.23	16.51	1.78
Swaziland (Eswatini)	DEV	14.55	17.96	15.83	1.3
Brazil	DEV	2.04	42.48	15.8	2.13
Tunisia	DEV	9.88	23.65	14.9	1.16
Cameroon	DEV	11.78	60.99	14.71	0.87
Nicaragua	DEV	3.62	32.61	13.28	1.52
Republic of Korea	DEV	10.2	14.53	13.21	0.86
Peru	DEV	4.9	18.36	11.94	1.14
Serbia	TRA	5.54	59.99	11.88	1.08
Russia	TRA	1.84	42.98	11.64	2.18
El Salvador	DEV	0	30.64	10.95	1.16
North Macedonia	TRA	5.29	53.09	10.68	1.33
Gabon	DEV	7.46	12.67	10.57	0.95
Belize	DEV	0	10.72	9.61	0.85
Ecuador	DEV	7.14	12.9	9.48	2.09
Venezuela	DEV	5.91	42.45	9.46	1.82
Pakistan	DEV	2.12	35.93	9.12	2.11
Colombia	DEV	5.34	18.36	9.06	1.95
Thailand	DEV	6.06	152.73	8.79	7.06
Romania	DPD	6.63	11.97	8.65	0.58
Croatia	DPD	3.95	34.13	8.65	2

Namibia	DEV	3.84	78.59	7.59	0.68
Ukraine	TRA	4.77	19.5	7.54	0.45
Bulgaria	DPD	5.29	23.08	7.42	0.45
Portugal	DPD	1.8	7.67	7.01	0.66
Belarus	TRA	4.95	9.62	6.8	0.3
Montenegro	TRA	4.57	59.99	6.77	3.27
Malaysia	DEV	5	8.34	6.77	0.45
Kazakhstan	TRA	5.88	44.49	6.58	0.24
Estonia	DPD	4.78	9.62	6.58	0.43
Ghana	DEV	3.88	26.92	6.52	0.78
Slovakia	DPD	3.01	7.41	6.27	0.21
Israel	DEV	4.19	41.34	6.1	2.14
Greece	DPD	5.29	54.15	5.63	1.24
Latvia	DPD	4.78	9.62	5.58	0.33
Lithuania	DPD	5.14	8.54	5.47	0.26
Bolivia	DEV	2.24	17.26	5.38	0.77
Jordan	DEV	0	44.42	5.26	0.85
Panama	DEV	3.63	9.84	5.16	1.12
Sri Lanka	DEV	3.5	7.97	5.13	0.59
South Africa	DEV	3.22	143.51	4.94	0.59
Chile	DEV	2.15	6.5	4.93	0.65
Mexico	DEV	0	9.41	4.9	0.44
Poland	DPD	2.22	8.54	4.88	0.33
Oman	DEV	3.48	49.72	4.84	0.27
Hungary	DPD	4.7	7.41	4.75	0.21
Saudi Arabia	DEV	1.62	54.75	4.64	0.78
Slovenia	DPD	2.85	8.53	4.54	0.92
Norway	DPD	1.12	14.47	4.46	1.18
Uruguay	DEV	4.08	12.44	4.27	0.15
Costa Rica	DEV	3.55	14.61	4.08	0.36
Switzerland	DPD	2.62	4.47	4.08	0.32
Zambia	DEV	2.38	202.28	3.47	1.06
Paraguay	DEV	1.5	14.65	3.45	0.46
Czechia	DPD	2.35	6.31	3.26	0.7
Italy	DPD	2.62	4.41	3.18	0.16
India	DEV	0.44	56.35	2.97	2.12
Austria	DPD	2.57	4.41	2.74	0.11
France	DPD	1.72	4.02	2.71	0.12
Argentina	DEV	1.81	18.36	2.31	0.17
Germany	DPD	2.22	5.08	2.3	0.18
Finland	DPD	1.35	14.47	2.11	0.18
Turkmenistan	TRA	1.93	35.82	2.06	0.41
Spain	DPD	1.43	7.67	1.92	0.2
Australia	DPD	1.11	2.26	1.91	0.2

United States	DPD	1.09	5.32	1.71	0.26
Canada	DPD	1.09	2.13	1.53	0.35
UAE	DEV	1.26	1.62	1.48	0.13
Sweden	DPD	1.12	6.85	1.29	0.1
Nepal	DEV	0.44	56.35	0.85	4.18
Qatar	DEV	0	5.18	0.11	0.74
Syria	DEV	0	40.31	0.02	0.41

Supplementary Table 3: Countries and international territories excluded from our analysis either due to a lack of carnivore presence within their borders, or due to a lack of cattle price data.

Countries excluded due to a lack of large carnivore presence	ISO3 Code	Economic Classification
Aland	ALA	DPD
Antigua and Barbuda	ATG	DEV*
Baker Island	UM	DPD
Bahamas	BHS	DPD
Bahrain	BHR	DEV
Barbados	BRB	DEV
Belgium	BEL	DPD
Bermuda	BMU	DPD
Brunei	BRN	DEV
Cabo Verde	CPV	DEV
Comoros	COM	DEV
Cuba	CUB	DEV
Cyprus	CYP	DPD
Denmark	DNK	DPD
Dominica	DMA	DEV*
Dominican Republic	DOM	DEV
Fiji	FJI	DEV
Grenada	GRD	DEV*
Haiti	HTI	DEV
Holy See	VAT	DPD
Hong Kong	HKG	DPD
Iceland	ISL	DPD
Ireland	IRL	DPD
Jamaica	JAM	DEV
Japan	JPN	DPD
Kiribati	KIR	DEV
Lichtenstein	LIE	DPD
Tonga	TON	DEV*
Tuvalu	TUV	DEV*
Taiwan	TWN	DPD
Madagascar	MDG	DEV
Maldives	MDV	DEV
Malta	MLT	DPD
Marshall Islands	MHL	DEV*
Mauritius	MUS	DEV
Micronesia	FSM	DEV
Monaco	MCO	DPD

Nauru	NRU	DEV*
Netherlands	NLD	DPD
Palau	PLW	DEV*
Papua New Guinea	PNG	DEV
Philipines	PHL	DEV
Saint Kitts and Nevis	KNA	DEV*
Saint Lucia	LCA	DEV*
Samoa	WSM	DEV*
San Marino	SMR	DPD
Sao Tome and Principe	STP	DEV
Seychelles	SYC	DEV*
Singapore	SGP	DPD
Solomon Islands	SLB	DEV
St Vincent and Grenadines	VCT	DEV*
Timor-Leste	TLS	DEV
Tonga	TON	DEV*
Trinidad and Tobago	TTO	DEV
Tuvalu	TUV	DEV*
United Kingdom	UK	DPD
Vanuatu	VUT	DEV*
Greenland	GRL	DPD
New Zealand	NZL	DPD
**Countries excluded due to no cattle price data**	**ISO3 Code**	**Economic Classification**
Andorra	AND	DPD
French Guiana	GF	DEV
Guatemala	GTM	DEV
Libya	LBY	DEV
DPRK (North Korea)	PRK	DEV
Lebanon	LBN	DEV
Somalia	SOM	DEV
Liberia	LBR	DEV

*Note: Economies not systematically monitored for the World Economic Situation and Prospects report.

Supplementary Table 4: Per capita calorie losses per year from the loss of a single cow and the total yearly protein calories from bovine meat after a loss is incurred per country. Data are taken from FAO Food Balances Database for the 2010 year: https://www.fao.org/faostat/en/#data/FBS (note these data only refer to bovine meat). We also provide prevalence of undernourishment for reader reference from the 2013 FAO State of Food Insecurity in the World Report: https://www.fao.org/3/i3434e/i3434e.pdf

**Country**	**Economic classification**	**Per capita protein lost from single cow or bull (g/day)**	**Per capita protein intake (g/day)**	**% per capita protein consumed relative to produced in a cow or bull**	**Kilocalories lost from single cow or bull***
Australia	DPD	686.03	14.66	2.14	806.29
Austria	DPD	886.03	6.95	0.78	1041.35
Bulgaria	DPD	406.85	1.84	0.45	478.17
Canada	DPD	916.44	10.91	1.19	1077.09
Croatia	DPD	458.36	3.68	0.80	538.71
Czechia	DPD	783.56	3.45	0.44	920.92

Estonia	DPD	590.14	4.98	0.84	693.59
Finland	DPD	824.66	7.67	0.93	969.22
France	DPD	805.21	10.22	1.27	946.36
Germany	DPD	859.45	4.52	0.53	1010.11
Greece	DPD	567.95	7.22	1.27	667.51
Hungary	DPD	709.59	1.83	0.26	833.98
Italy	DPD	754.25	9.35	1.24	886.47
Latvia	DPD	496.16	2.14	0.43	583.14
Lithuania	DPD	606.30	1.58	0.26	712.59
Norway	DPD	743.29	6.72	0.90	873.59
Poland	DPD	651.51	0.84	0.13	765.72
Portugal	DPD	632.60	6.98	1.10	743.50
Romania	DPD	446.58	3.08	0.69	524.86
Slovakia	DPD	694.52	1.67	0.24	816.27
Slovenia	DPD	771.23	7.53	0.98	906.43
Spain	DPD	710.68	5.4	0.76	835.27
Sweden	DPD	833.70	9.12	1.09	979.85
Switzerland	DPD	598.90	7.45	1.24	703.89
United States	DPD	961.64	13.1	1.36	1130.22
Albania	TRA	301.92	6.22	2.06	354.84
Armenia	TRA	309.86	8.09	2.61	364.18
Azerbaijan	TRA	305.48	5.13	1.68	359.03
Belarus	TRA	469.86	8.27	1.76	552.23
Bosnia and Herzegovina	TRA	438.36	3.82	0.87	515.20
Georgia	TRA	196.71	3.07	1.56	231.20
Kazakhstan	TRA	427.40	9.57	2.24	502.32
Kyrgyzstan	TRA	479.73	5.68	1.18	563.82
Montenegro	TRA	476.99	5.22	1.09	560.60
North Macedonia	TRA	370.68	3.54	0.95	435.67
Republic of Moldova	TRA	356.16	0.76	0.21	418.60
Russia	TRA	507.40	6.91	1.36	596.34
Serbia	TRA	611.51	3.83	0.63	718.70
Tajikistan	TRA	484.38	1.45	0.30	569.30
Turkmenistan	TRA	493.15	10.12	2.05	579.60
Ukraine	TRA	421.92	3.67	0.87	495.88
Uzbekistan	TRA	493.15	10.17	2.06	579.60
Afghanistan	DEV	493.15	1.89	0.38	579.60
Algeria	DEV	533.70	1.91	0.36	627.26
Angola	DEV	465.75	2.4	0.52	547.40
Argentina	DEV	576.71	25.4	4.40	677.81
Bangladesh	DEV	193.42	0.55	0.28	227.33

Belize	DEV	559.18	1.86	0.33	657.20
Benin	DEV	487.95	1.5	0.31	573.48
Bhutan	DEV	282.19	-	-	331.66
Bolivia	DEV	364.11	7.35	2.02	427.94
Botswana	DEV	547.95	2.76	0.50	644.00
Brazil	DEV	620.27	14.36	2.32	729.01
Burkina Faso	DEV	309.59	3.17	1.02	363.86
Burundi	DEV	547.95	0.46	0.08	644.00
Cambodia	DEV	328.77	2.06	0.63	386.40
Cameroon	DEV	397.81	1.63	0.41	467.54
Chad	DEV	328.77	9.41	2.86	386.40
Chile	DEV	663.01	8.09	1.22	779.24
China	DEV	400.00	1.74	0.44	470.12
Colombia	DEV	580.00	6.3	1.09	681.67
Congo	DEV	429.04	1.06	0.25	504.25
Costa Rica	DEV	633.15	7.24	1.14	744.14
DRC	DEV	426.85	0.11	0.03	501.68
Djibouti	DEV	301.37	2.98	0.99	354.20
Ecuador	DEV	558.90	6.63	1.19	656.88
Egypt	DEV	866.03	4.49	0.52	1017.84
El Salvador	DEV	453.42	4.01	0.88	532.91
Equatorial Guinea	DEV	301.37	-	-	354.20
Eritrea	DEV	273.97	-	-	322.00
Ethiopia	DEV	299.18	1.93	0.65	351.62
Gabon	DEV	361.64	2.55	0.71	425.04
Gambia	DEV	328.77	0.93	0.28	386.40
Ghana	DEV	342.47	0.5	0.15	402.50
Guinea	DEV	255.89	2.47	0.97	300.75
Guinea-Bissau	DEV	301.37	1.68	0.56	354.20
Guyana	DEV	477.81	1.28	0.27	561.57
Honduras	DEV	489.04	3.07	0.63	574.77
India	DEV	282.19	0.52	0.18	331.66
Indonesia	DEV	617.81	0.96	0.16	726.11
Iran	DEV	621.37	3.29	0.53	730.30
Iraq	DEV	578.08	1.15	0.20	679.42
Israel	DEV	959.45	11.26	1.17	1127.64
Ivory Coast	DEV	296.16	0.79	0.27	348.08
Jordan	DEV	547.95	1.85	0.34	644.00
Kenya	DEV	643.29	4.39	0.68	756.06
Laos	DEV	342.47	2.45	0.72	402.50
Lesotho	DEV	356.16	2.15	0.60	418.60

Malawi	DEV	340.00	0.89	0.26	399.60
Malaysia	DEV	923.56	2.37	0.26	1085.46
Mali	DEV	356.16	3.98	1.12	418.60
Mauritania	DEV	328.77	2.92	0.89	386.40
Mexico	DEV	564.38	6.83	1.21	663.32
Mongolia	DEV	310.96	5.71	1.84	365.47
Morocco	DEV	530.68	2.67	0.50	623.71
Mozambique	DEV	273.97	0.21	0.08	322.00
Myanmar	DEV	584.66	2.07	0.35	687.15
Namibia	DEV	676.44	3.99	0.59	795.02
Nepal	DEV	232.88	2.54	1.09	273.70
Nicaragua	DEV	443.56	1.27	0.29	521.32
Niger	DEV	1045.75	1.47	0.14	1229.07
Nigeria	DEV	356.16	0.78	0.22	418.60
Oman	DEV	356.16	4.61	1.29	418.60
Pakistan	DEV	533.42	2.96	0.55	626.93
Palestine	DEV	561.37	-	-	659.78
Panama	DEV	594.52	7.76	1.31	698.74
Paraguay	DEV	695.62	4.6	0.66	817.56
Peru	DEV	386.30	2.5	0.65	454.02
Qatar	DEV	328.77	-	-	386.40
Republic of Korea	DEV	896.44	4.33	0.48	1053.58
Rwanda	DEV	284.93	1.07	0.38	334.88
Saudi Arabia	DEV	547.95	2.41	0.44	644.00
Senegal	DEV	406.03	2.33	0.57	477.20
Sierra Leone	DEV	282.19	0.5	0.18	331.66
South Africa	DEV	765.75	6.99	0.91	899.99
Sri Lanka	DEV	372.88	0.67	0.18	438.24
Sudan (former)	DEV	332.60	3.49	1.05	390.91
Suriname	DEV	483.29	4.11	0.85	568.01
Swaziland (Eswatini)	DEV	620.55	7.21	1.16	729.33
Syria	DEV	410.96	1.42	0.35	483.00
Tanzania	DEV	262.47	2.19	0.83	308.48
Thailand	DEV	680.27	1.06	0.16	799.53
Togo	DEV	342.47	0.62	0.18	402.50
Tunisia	DEV	465.75	2.26	0.49	547.40
Turkey	DEV	593.42	4.51	0.76	697.45
UAE	DEV	684.93	-	-	805.00
Uganda	DEV	409.86	2.22	0.54	481.71
Uruguay	DEV	570.96	11.46	2.01	671.05
Venezuela	DEV	657.53	6.19	0.94	772.80

Vietnam	DEV	471.51	1.9	0.40	554.16
Yemen	DEV	249.32	2.14	0.86	293.02
Zambia	DEV	438.36	4.53	1.03	515.20
Zimbabwe	DEV	615.62	3.58	0.58	723.53

Note: Protein intake data are from the FAO food balances database for the 2010 year. Burundi data are from 2014 while Sudan is from 2012.

*For the calculation of calories, we took beef carcass kilojoule value (1351kj/100g) from the FAO (https://www.fao.org/ag/againfo/themes/en/meat/backgr_composition.html)

Supplementary References

1.Gusset, M., Swarner, M. J., Mponwane, L., Keletile, K., & McNutt, J. W. (2009). Human–wildlife conflict in northern Botswana: livestock predation by endangered African wild dog Lycaon pictus and other carnivores. *Oryx*, *43*(1), 67-72.

2.Doan-Crider, D. L., Tri, A. N., & Hewitt, D. G. (2017). Woody cover and proximity to water increase American black bear depredation on cattle in Coahuila, Mexico. *Ursus*, *28*(2), 208-217.

3.Liu, F., McShea, W. J., Garshelis, D. L., Zhu, X., Wang, D., & Shao, L. (2011). Human-wildlife conflicts influence attitudes but not necessarily behaviors: Factors driving the poaching of bears in China. *Biological Conservation*, *144*(1), 538-547.

4.Anderson Jr, C. R., Ternent, M. A., & Moody, D. S. (2002). Grizzly bear-cattle interactions on two grazing allotments in northwest Wyoming. *Ursus*, 247-256.
5.Schiess-Meier, M., Ramsauer, S., Gabanapelo, T., & König, B. (2007). Livestock Predation-Insights From Problem Animal Control Registers in Botswana. *The Journal of Wildlife Management*, 1267-1274.

6.Voigt, C. C., Thalwitzer, S., Melzheimer, J., Blanc, A. S., Jago, M., & Wachter, B. (2014). The conflict between cheetahs and humans on Namibian farmland elucidated by stable isotope diet analysis. *PLoS One*, *9*(8), e101917.

7.Wang, S. W., & Macdonald, D. W. (2009). Feeding habits and niche partitioning in a predator guild composed of tigers, leopards and dholes in a temperate ecosystem in central Bhutan. *Journal of Zoology*, *277*(4), 275-283.

8.Doherty, T. S., Davis, N. E., Dickman, C. R., Forsyth, D. M., Letnic, M., Nimmo, D. G., ... & Newsome, T. M. (2019). Continental patterns in the diet of a top predator: Australia's dingo. *Mammal Review*, *49*(1), 31-44.

9.Stahler, D. R., Smith, D. W., & Guernsey, D. S. (2006). Foraging and feeding ecology of the gray wolf (Canis lupus): lessons from Yellowstone National Park, Wyoming, USA. *The Journal of nutrition*, *136*(7), 1923S-1926S.

10.Rosas-Rosas, O. C., Bender, L. C., & Valdez, R. (2008). Jaguar and puma predation on cattle calves in northeastern Sonora, Mexico. *Rangeland Ecology & Management*, *61*(5), 554-560.

11.Hayward, M. W., Henschel, P., O'Brien, J., Hofmeyr, M., Balme, G., & Kerley, G. I. (2006). Prey preferences of the leopard (Panthera pardus). *Journal of Zoology*, *270*(2), 298-313.

12.Bauer, H., & De Iongh, H. H. (2005). Lion (Panthera leo) home ranges and livestock conflicts in Waza National Park, Cameroon. *African journal of ecology*, *43*(3), 208-214.

13.Palmeira, F. B., Crawshaw Jr, P. G., Haddad, C. M., Ferraz, K. M. P., & Verdade, L. M. (2008). Cattle depredation by puma (Puma concolor) and jaguar (Panthera onca) in central-western Brazil. *Biological conservation*, *141*(1), 118-125.

14.Lyngdoh, S., Shrotriya, S., Goyal, S. P., Clements, H., Hayward, M. W., & Habib, B. (2014). Prey preferences of the snow leopard (Panthera uncia): regional diet specificity holds global significance for conservation. *PloS one*, *9*(2), e88349.

15.Kissui, B. M. (2008). Livestock predation by lions, leopards, spotted hyenas, and their vulnerability to retaliatory killing in the Maasai steppe, Tanzania. *Animal conservation*, *11*(5), 422-432.

16.Bhandari, S., Morley, C., Aryal, A., & Shrestha, U. B. (2020). The diet of the striped hyena in Nepal's lowland regions. *Ecology and Evolution*, *10*(15), 7953-7962.

17.Sangay, T., & Vernes, K. (2008). Human–wildlife conflict in the Kingdom of Bhutan: patterns of livestock predation by large mammalian carnivores. *Biological Conservation*, *141*(5), 1272-1282.

18.Consorte-McCrea, A., & Santos, E. F. (2013). *Ecology and Conservation of the Maned Wolf*. London: CRC Press.

19.Chiang, P. J., & Allen, M. L. (2017). A review of our current knowledge of clouded leopards (Neofelis nebulosa). *arXiv preprint arXiv:1712.04377*.
