# Supplementary material for: The unequal burden of human-wildlife conflict
Source: Commun Biol. 2023 Feb 23;6:182. doi: 10.1038/s42003-023-04493-y (PMC9950466; doi:10.1038/s42003-023-04493-y)
Supplement: Supplementary file 3 — Description of Additional Supplementary Files [file 42003_2023_4493_MOESM3_ESM.pdf]

Description of Additional Supplementary Files

File name: Supplementary Data 1
Description: Cattle prices and GDP Data. All the official FAO cattle price data as of 21 June 2021 (date of download). This spreadsheet includes all of the raw cattle price data as provided by the FAO, the online sources of data and a description of where data was obtained if FAO data could not be sourced for 2009 (AOH carnivore data year).

File name: Supplementary Data 2
Description: Meat Yield Data. Shows meat yield over time and for the 2009 year. For data on meat yield per animal we used FAO data downloaded from: https://www.fao.org/faostat/en/#search/cattle%20867 – data are presented as kilograms of meat per animal (minus offal). The specific name of the data on the FAO website is "Meat, cattle (Item) Crops and livestock products (Production)". Data span a time series from 1961 – 2020 (or next best year).

File name: Supplementary Data 3
Description: Economic burden Data. Raw analysed data demarcating our 5 income quantiles and the proportion of range that is found within each of these for 18 large carnivore species. It also contains development categories according to economic status of each country (as well as mean and std deviation). Final n = 133 countries.
