# Supplementary material for: The unequal burden of human-wildlife conflict
Source: Commun Biol. 2023 Feb 23;6:182. doi: 10.1038/s42003-023-04493-y (PMC9950466; doi:10.1038/s42003-023-04493-y)
Supplement: Supplementary file 7 — Reporting Summary [file 42003_2023_4493_MOESM7_ESM.pdf]

nature portfolio

Corresponding author(s): Luke Gibson
Last updated by author(s): Dec 15, 2022

Reporting Summary

Nature Portfolio wishes to improve the reproducibility of the work that we publish. This form provides structure for consistency and transparency in reporting. For further information on Nature Portfolio policies, see our Editorial Policies and the Editorial Policy Checklist.

Statistics

For all statistical analyses, confirm that the following items are present in the figure legend, table legend, main text, or Methods section.

n/a	Confirmed	
☐	☒	The exact sample size (*n*) for each experimental group/condition, given as a discrete number and unit of measurement
☐	☒	A statement on whether measurements were taken from distinct samples or whether the same sample was measured repeatedly
☐	☒	The statistical test(s) used AND whether they are one- or two-sided<br>*Only common tests should be described solely by name; describe more complex techniques in the Methods section.*
☒	☐	A description of all covariates tested
☒	☐	A description of any assumptions or corrections, such as tests of normality and adjustment for multiple comparisons
☐	☒	A full description of the statistical parameters including central tendency (e.g. means) or other basic estimates (e.g. regression coefficient) AND variation (e.g. standard deviation) or associated estimates of uncertainty (e.g. confidence intervals)
☐	☒	For null hypothesis testing, the test statistic (e.g. *F*, *t*, *r*) with confidence intervals, effect sizes, degrees of freedom and *P* value noted<br>*Give P values as exact values whenever suitable.*
☒	☐	For Bayesian analysis, information on the choice of priors and Markov chain Monte Carlo settings
☒	☐	For hierarchical and complex designs, identification of the appropriate level for tests and full reporting of outcomes
☒	☐	Estimates of effect sizes (e.g. Cohen's *d*, Pearson's *r*), indicating how they were calculated

Our web collection on statistics for biologists contains articles on many of the points above.

Software and code

Policy information about availability of computer code

Data collection	All our data is available openly and is provided as part of this manuscript (excepting the AOH data from Rondinini et al. 2011 which was made available upon request).
Data analysis	We performed our spatial analyses in ESRI ArcGIS V10.8 while analysis of variance were performed online using https://www.socscistatistics.com/tests/anova/default2.aspx and https://www.statskingdom.com/180Anova1way.html

For manuscripts utilizing custom algorithms or software that are central to the research but not yet described in published literature, software must be made available to editors and reviewers. We strongly encourage code deposition in a community repository (e.g. GitHub). See the Nature Portfolio guidelines for submitting code & software for further information.

Data

Policy information about availability of data

All manuscripts must include a data availability statement. This statement should provide the following information, where applicable:
- Accession codes, unique identifiers, or web links for publicly available datasets
- A description of any restrictions on data availability
- For clinical datasets or third party data, please ensure that the statement adheres to our policy

We provide all used data as excel spreadsheets saved as the following:

1. Cattle prices and GDP_Data 1.xslx
2. Meat Yield_Data 2.xlsx
3. Economic burden_Data 3.xlsx

The "Cattle prices and GDP.xlsx" contains all the official FAO cattle price data as of 21 June 2021 (date of download). This spreadsheet includes all of the raw cattle price data as provided by the FAO, the online sources of data and a description of where data was obtained if FAO data could not be sourced for 2009 (AOH carnivore data year). "Meat Yield_Data 2.xslx" shows meat yield over time and for the 2009 year. For data on meat yield per animal we used FAO data downloaded from: https://www.fao.org/faostat/en/#search/cattle%20867 – data are presented as kilograms of meat per animal (minus offal). The specific name of the data on the FAO website is "Meat, cattle (Item) Crops and livestock products (Production)". Data span a time series from 1961 – 2020 (or next best year). The " Economic burden_Data 3.xslx" contains the raw analysed data demarcating our 5 income quantiles and the proportion of range that is found within each of these for 18 large carnivore species. It also contains development categories according to economic status of each country (as well as mean and std deviation). Final n = 133 countries.

Human research participants

Policy information about studies involving human research participants and Sex and Gender in Research.

Reporting on sex and gender	*Use the terms sex (biological attribute) and gender (shaped by social and cultural circumstances) carefully in order to avoid confusing both terms. Indicate if findings apply to only one sex or gender; describe whether sex and gender were considered in study design whether sex and/or gender was determined based on self-reporting or assigned and methods used. Provide in the source data disaggregated sex and gender data where this information has been collected, and consent has been obtained for sharing of individual-level data; provide overall numbers in this Reporting Summary. Please state if this information has not been collected. Report sex- and gender-based analyses where performed, justify reasons for lack of sex- and gender-based analysis.*
Population characteristics	*Describe the covariate-relevant population characteristics of the human research participants (e.g. age, genotypic information, past and current diagnosis and treatment categories). If you filled out the behavioural & social sciences study design questions and have nothing to add here, write "See above."*
Recruitment	*Describe how participants were recruited. Outline any potential self-selection bias or other biases that may be present and how these are likely to impact results.*
Ethics oversight	*Identify the organization(s) that approved the study protocol.*

Note that full information on the approval of the study protocol must also be provided in the manuscript.

Field-specific reporting

Please select the one below that is the best fit for your research. If you are not sure, read the appropriate sections before making your selection.

☐ Life sciences ☐ Behavioural & social sciences ☒ Ecological, evolutionary & environmental sciences

For a reference copy of the document with all sections, see nature.com/documents/nr-reporting-summary-flat.pdf

Ecological, evolutionary & environmental sciences study design

All studies must disclose on these points even when the disclosure is negative.

Study description

Mapping large carnivore habitat
We mapped the spatial habitat extent of 18 large carnivores known to prey on cattle (Supplementary Table 1) using Area of Habitat (AOH) data from Rondinini et al. (2011). AOH represents areas of fine scale (300m) high habitat suitability within large carnivore IUCN geographic distributions during the year 2009. AOH has been used in several recent global studies (eg. O'Bryan et al. 2021; Strassburg et al. 2020) due to its reduced risk for commission errors (Brooks et al. 2019), fine spatial scale, and incorporation of heterogeneous environmental variables (eg. land cover, elevation, and hydrological features, Rondinini et al. 2006). The AOH layers for each species were used for subsequent analyses.

Mapping per capita income
We mapped annual per capita income at the sub-national scale (i.e., state or province scale) from Lessman and Seidel (2017) for the year 2009. We chose the year 2009 to correspond with the year of the large carnivore AOH data, as described above. We chose the Lessman and Seidel (2017) dataset because these data provide high resolution estimates of per capita income for both developed and developing countries based on nighttime luminosity data. Nighttime luminosity is important because household survey data (e.g., Afrobarometer or DHS) usually do not contain income data since households fear expropriation from the government and/or cannot provide a monetary equivalent of returns from agricultural production. Generally, as income rises, so too does electricity usage and subsequent nighttime light signature per person, in both production activities and consumptive ones (Henderson et al. 2012), and light has been used as a proxy for income per capita in several previous studies (e.g., Ebener et al. 2005; Chen et al. 2011; Jean et al. 2016).

Estimating cattle prices
We obtained data on national cattle prices per kilogram (Item Code 945, FAO) from the Food and Agriculture Organization of the

United Nations (FAO 2021). This dataset reports on cattle prices as collected at the point of initial sale (prices paid at the farm-gate). Because our large carnivore AOH and per capita income data corresponded to the year 2009, we included the average cattle live weight price for the year 2009, or the next closest year available in the FAO database or grey literature (see Supplementary Data File 1 for details). We then determined the price of a single sub-adult cattle calf by multiplying the per kilogram cattle price for a given country by 250 kg, the approximate size of a sub-adult cattle calf, also known as a single tropical livestock unit (Lybbert et al. 2004). This estimated price of a sub-adult cattle calf per country was used for estimating the financial burden of large carnivore predation, described below.

Mapping cattle distribution
We mapped the spatial distribution of cattle using the updated Gridded Livestock of the World (GLW 3; Gilbert et al. 2018). The GLW 3 represents areas of sub-national livestock densities, including cattle at fine resolution (approximately 10km at the equator) for the year 2010. We used the dasymetric dataset of cattle, which corresponds to previous GLW datasets and represents different cattle densities per pixel within a census area according to random forest models. We determine cattle to be present in a given pixel if the density was greater than zero. We resampled the data (bilinear method) to match the spatial resolution of the large carnivore AOH data (300m).

Mapping burden hotspots
We determined the financial burden of losing a single sub-adult cattle calf to large carnivore predation by firstly dividing estimates of the price of a single sub-adult cattle calf (see Estimating cattle prices) with estimates of per capita income at the sub-national scale (see Estimating per capita income). This produced the relative proportion of annual per capita income lost assuming the predation of a single sub-adult cattle calf. We then masked sub-national administrative boundaries, which contained the above information on the proportion of per capita income vulnerable, with the extent of large carnivore AOH (see Mapping large carnivore habitat) – that is a spatial mosaic of all 18 large carnivore AOH. Next, we masked this layer with data from Gilbert et al. (2018) on the spatial distribution of cattle globally (see Mapping cattle distribution). Finally, we intersected the AOH (corrected with the distribution of cattle) with country boundaries to determine the per capita financial burden at the national scale, and we intersected this with individual carnivore AOH layers to determine the potential financial burden within each species' geographic range.

We report our results of potential economic burden for each country as an average annual per capita income loss across the entire country. We also calculated the proportion of each carnivore species' range overlapping areas which experience different levels of economic burden: 0-5% of per capita income loss (very low-vulnerability area), 5-10% (low vulnerability area), 10-25% (moderate vulnerability area), 25-50% (high vulnerability area), and >50% (extreme vulnerability area).

Estimating food security impacts
We also assessed the direct and opportunity costs lost to households from predation. We calculated calories lost from a single cattle calf predation through multiplying country-specific meat yield (FAO 2021) by beef carcass kilojoule value (1351kj/100g) from the FAO (see: https://www.fao.org/ag/againfo/themes/en/meat/backgr_composition.html). We then divided these lost calories by the average estimated daily caloric intake of a young child aged 2-3, an adolescent aged 12-13, and an adult aged 30-60-years-old (United Nations University and World Health Organization 2004).

Estimating economic disparities in national cattle production
Finally, we assessed the disparities in cattle prices between developing, transition, and developed economies and examined the differences in meat yield per carcass. We did this because we wanted to ascertain whether pastoralists in developing and transition economies may be further exposed (comparatively to developed states) to conflict due to a) price plasticity in cattle markets over time (these could be indicative of market or climate-related shocks, Maystadt and Ecker 2014, Lybbert et al. 2004), and b) low productivity in meat production per animal (ie. prices of cattle per kg may be similar between countries however pastoralists have to produce more cattle per unit area to yield the same price per ton). We used cattle meat yields in kilograms per animal obtained from the FAO agricultural database (FAO 2021). Potential disparities in a) the cattle price (both for the 2009 year, and over time using our historic FAO cattle price dataset), and b) meat yield per animal between developing, transitioning, and developed economies were assessed using a one-way analysis of variance ANOVA.

Research sample	For mapping burden hot spots we used only carnivores known to prey on cattle. We used cattle price data from the FAO (FAO Meat live weight, cattle database (2021). License: CC BY-NC-SA 3.0 IGO. Extracted from: http://www.fao.org/faostat/en/#search/cattle. Data of Access: 24-04-2021), and used per capita income generated from nighttime light data (Lessmann and Seidel 2017).
Sampling strategy	Because our large carnivore AOH corresponded to the year 2009, we included the average cattle live weight price for the year 2009, next closest year available in the UN database or an appropriate source found in unpublished reports, agricultural catalogues, or other online resources.
Data collection	All data on cattle prices and weights were taken from FAO (2021) and are publicly available/attached to this paper.
Timing and spatial scale	AOH carnivore data are for 2009 as are FAO cattle price data (mapping burden hot spots) and per capita income measures.
Data exclusions	Countries and international territories excluded from our analysis were done so either due to a lack of carnivore presence within their borders, or due to a lack of cattle price data. Species omitted from our analysis due to no evidence found in the literature for predation on cattle: Clouded leopard Neofelis nebulosa, Eurasian lynx Lynx lynx, Maned wolf Chrysocyon brachyurus, and Ethiopian wolf Canis simensis due to no evidence being found in the available peer-reviewed literature of these species consuming cattle in their diet (Consorte-McCrea 2013, Chiang and Allen 2017).
Reproducibility	All attempts to repeat the experiment were successful.
Randomization	N/A

Blinding	N/A

Did the study involve field work? ☐ Yes ☒ No

Reporting for specific materials, systems and methods

We require information from authors about some types of materials, experimental systems and methods used in many studies. Here, indicate whether each material, system or method listed is relevant to your study. If you are not sure if a list item applies to your research, read the appropriate section before selecting a response.

Materials & experimental systems

n/a	Involved in the study
☒	☐ Antibodies
☒	☐ Eukaryotic cell lines
☒	☐ Palaeontology and archaeology
☐	☒ Animals and other organisms
☒	☐ Clinical data
☒	☐ Dual use research of concern

Methods

n/a	Involved in the study
☒	☐ ChIP-seq
☒	☐ Flow cytometry
☒	☐ MRI-based neuroimaging

Animals and other research organisms

Policy information about studies involving animals; ARRIVE guidelines recommended for reporting animal research, and Sex and Gender in Research

Laboratory animals	N/A
Wild animals	N/A
Reporting on sex	N/A
Field-collected samples	N/A
Ethics oversight	N/A

Note that full information on the approval of the study protocol must also be provided in the manuscript.
